# Seroprevalence and Spatial Distribution of Hepatitis C Virus in Bahia, Brazil

**DOI:** 10.4269/ajtmh.20-1615

**Published:** 2021-08-23

**Authors:** Felicidade Mota Pereira, Fred Luciano Neves Santos, Maria da Conceição Chagas de Almeida, Roberto Perez Carreiro, Luciano Kalabric Silva, Bernardo Galvão-Castro, Maria Fernanda Rios Grassi

**Affiliations:** ^1^ Advanced Public Health Laboratory, Gonçalo Moniz Institute, Fiocruz-BA, Salvador, Bahia, Brazil;; ^2^ Gonçalo Moniz Public Health Central Laboratory, Salvador, Bahia, Brazil;; ^3^ Molecular Epidemiology and Biostatistics Laboratory, Gonçalo Moniz Institute, Fiocruz-BA, Salvador, Bahia, Brazil;; ^4^ Center for Integration of Data and Health Knowledge, Gonçalo Moniz Institute, Fiocruz-BA, Salvador, Bahia, Brazil;; ^5^ Bahiana School of Medicine and Public Health, Salvador, Bahia, Brazil

## Abstract

In Salvador, which is the capital of the Brazilian state of Bahia, it has been estimated that 1.5% of the general population is infected with hepatitis C virus (HCV); however, the circulation of HCV throughout the state remains unknown. The present retrospective study aimed to determine anti-HCV seroprevalence and describe the geographic distribution of hepatitis C in Bahia. Data from HCV serological tests submitted to the Bahia Central Laboratory of Public Health between 2004 and 2013 were analyzed. Serology for HCV was performed using the AxSYM anti-HCV enzymatic microparticle immunoassay and chemiluminescence immunoassay. A subgroup of samples with detectable HCV-RNA was genotyped using the linear array hepatitis C virus genotyping assay. A total of 247,837 samples were analyzed. The median age of the studied population was 31 years (interquartile range, 25–44 years), and the female:male ratio was 3.9:1. The global seroprevalence of HCV in Bahia was estimated to be 1.3% (3,230/247,837), corresponding to an infection rate of 21.2/100,000 inhabitants. The seroprevalence of HCV was higher among males and increased with age. The presence of anti-HCV antibodies was detected throughout all mesoregions of Bahia, and the municipality with the highest infection rate was Ipiaú (112.04 cases/100,000 inhabitants). Genotypes 1 and 3 were found to be the most prevalent, followed by genotypes 2, 4, and 5. Our results provide evidence of the widespread distribution of previous HCV infection throughout the state of Bahia.

## INTRODUCTION

The hepatitis C virus (HCV) is mainly transmitted through the use of contaminated needles, syringes, and instruments used for injection and skin-piercing procedures.^1^ The sexual transmission of HCV is rare.^2^ The majority of cases of acute HCV infection progress to a chronic infection with an asymptomatic course. Approximately two to three decades after the onset of the infection, 10% to 20% of infected individuals will develop cirrhosis and 1% to 5% will develop hepatocellular carcinoma.^3^^,^^4^

It has been estimated that 71 million people worldwide are chronically infected with HCV. A significant number of chronically infected individuals are at risk for cirrhosis or liver cancer.^5^ In Brazil, a national population-based study conducted in the capitals estimated that the overall seroprevalence of anti-HCV antibodies is 1.38%.^6^ However, the prevalence of this infection varies according to the geographical region and group studied. The groups at most significant risk for infection are intravenous drug users,^7^ individuals undergoing predialysis,^8^ individuals with coagulation disorders, and individuals with chronic renal failure.^9^ Other groups such as health waste handlers,^10^ those undergoing hemodialysis,^11^ incarcerated individuals,^12^ and pregnant women^13^ have higher prevalence rates than individuals in the general population. Despite low frequencies of HCV infection, sex workers,^14^ Amerindians from six tribes in the Eastern Amazon region,^15^ and military personnel^16^ all have higher rates than blood donors.^17^

Accordingly, the Notifiable Diseases Information System (SINAN; Brazilian Ministry of Health) has indicated that northeast Brazil has the third highest number of HCV cases in the country. In 2017, the state of Bahia had a detection rate of anti-HCV or RNA-HCV of 4.5 cases per 100,000 inhabitants.^18^ In the city of Salvador, which is the capital of the state of Bahia, a population-based study reported a 1.5% prevalence of HCV infection.^19^ However, the circulation of HCV and genotypes throughout the microregions of Bahia remains unclear. This study aimed to determine the anti-HCV seroprevalence and describe the geographical distribution of HCV infection in the state of Bahia.

## MATERIALS AND METHODS

### Ethical statement.

The institutional review board (IRB) for Human Research at the Gonçalo Moniz Institute of the Oswaldo Cruz Foundation (Salvador, Bahia, Brazil) provided ethical approval to conduct this study (CAAE number 22478813.7.0000.0040).

### Study area.

**Figure 1.**
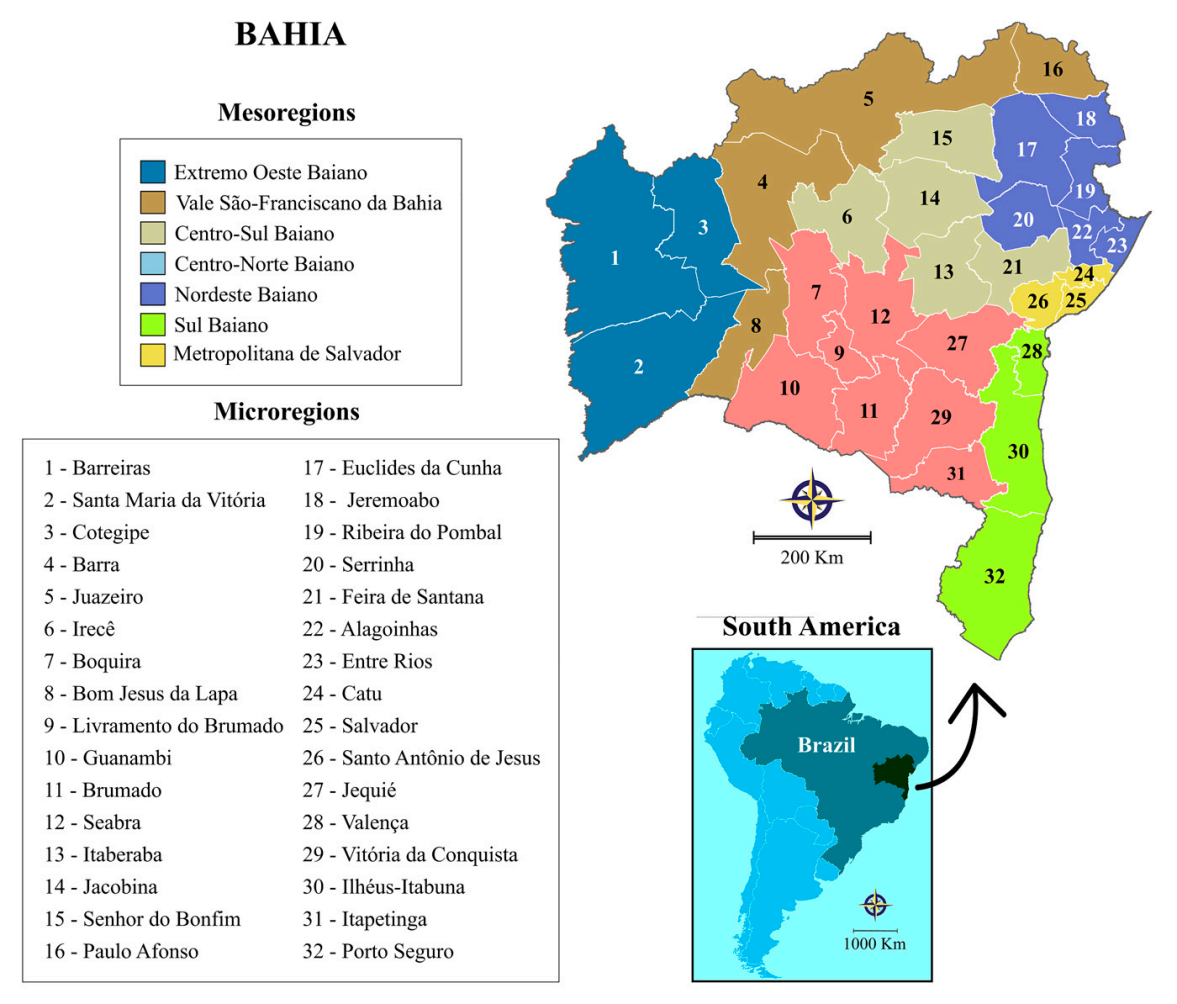
Geographic division of the state of Bahia into seven mesoregions and 32 microregions. Digital maps in the public domain were obtained from the Brazilian Institute of Geography and Statistics (IBGE) cartographic database in shapefile format (.shp); these were subsequently reformatted and analyzed using QGIS version 3.10 (Geographic Information System, Open-Source Geospatial Foundation Project; http://qgis.osgeo.org). This figure appears in color at www.ajtmh.org.

This study was conducted in the state of Bahia, which is the fourth most populous Brazilian state and the fifth largest area in the country (565,733 km^2^). In accordance with economic and social similarities among its 417 municipalities, the Brazilian National Institute of Geography and Statistics (IBGE) delineated seven mesoregions that are further grouped into 32 microregions (Figure 1). In 2015, the annual population estimate for Bahia was 15,203,934 inhabitants, resulting in an overall density of 27 inhabitants per square kilometer (http://www.ibge.gov.br).

### Study design.

A retrospective ecological study was conducted using data obtained from the Central Laboratory of Public Health of Bahia (LACEN-BA). All serological tests for HCV were selected among the 32 Bahia microregions from 2004 to 2013. The unique registration number of each sample was considered the key variable. To avoid duplication, the most recent serological results were considered. The target population comprised mainly pregnant women, blood donors, and individuals exhibiting symptoms of infectious disease referred by prenatal physicians, blood banks, or clinicians in the public health system.

### Laboratory testing.

Serological testing for HCV was performed using a microparticle enzyme immunoassay (MEIA; AxSYM Anti-HCV Abbott Diagnostics Division, Illinois, USA) until 2009; after that time, the chemiluminescent enzyme immunoassay (CLIA) (Architect Anti-HCV; Abbott Diagnostics Division, Wiesbaden, Germany) was implemented for HCV screening. For some MEIA/CLIA-positive samples, physicians ordered confirmation of HCV RNA by quantitative reverse-transcription polymerase chain reaction (AMPLICOR®; Roche Molecular Systems, Branchburg, NJ) if alterations in the clinical status and laboratory parameters were detected. Genotyping analysis, which was requested at the onset of treatment or if resistance was suspected, was performed by analyzing the highly conserved 5′ untranslated region using the Linear Array Hepatitis C Virus Genotyping Test (LiPA; Line Probe Assay, Roche Diagnostics, Indianapolis, IN) according to the manufacturer’s guidelines. This assay allows for the determination of six genotypes and subtypes (1a, 1b, 2, 2a, 2b, 3, 3a, 4, 4c, 5, 5a, and 6).

### Data analysis.

The SMART LABORATORY management system was used to extract data from all serological tests performed during each year of the study period. Validation of the sample database was performed using the R software package, and it was analyzed using STATA v. 13.0. Age is expressed as the median and interquartile range (IQR). Absolute and relative frequencies were calculated for the categorical variables of age (0–15 years, 16–30 years, 31–50 years, or 51 years or older), sex (male or female), and serological test (reagent or nonreagent). GraphPad Prism v. 7 (GraphPad Software, San Diego, CA) was used for data analysis; differences were considered statistically significant when *P* < 0.05. Rates of infection and coinfection were expressed as the number of individuals infected per 100,000 inhabitants. All HCV cases diagnosed on the municipality level and specifically linked to the municipality of residence of each HCV case were grouped into microregions and mesoregions, which were then used as units for the analyses and comparisons of the different regions to reveal priority areas for interventions. To estimate infection rates, population data were obtained from the Brazilian Institute of Geography and Statistics (IBGE) based on the national census for the period between 2000 and 2010 (https://sidra.ibge.gov.br/pesquisa/censo-demografico/series-temporais/series-temporais/). The official annual population estimates were used for the remaining years (available at https://sidra.ibge.gov.br/pesquisa/estimapop/tabelas). The 3-year moving averages were calculated between 2004 and 2013. The annual age-adjusted and sex-adjusted incidence rates with corresponding 95% confidence intervals (CIs) were calculated per 100,000 inhabitants using population census data from 2010 and annual population estimates. Maps were created using the Brazilian annual incidence at the beginning of the studied period as a denominator to illustrate the relative risk of HCV among the Bahia microregions. Mapping was performed using QGIS software version 3.10 (Geographic Information System, Open-Source Geospatial Foundation Project; freely available at http://qgis.osgeo.org). Digital maps were obtained from the IBGE database in shapefile (.shp) format, which is compatible with the QGIS program.

## RESULTS

**Figure 2.**
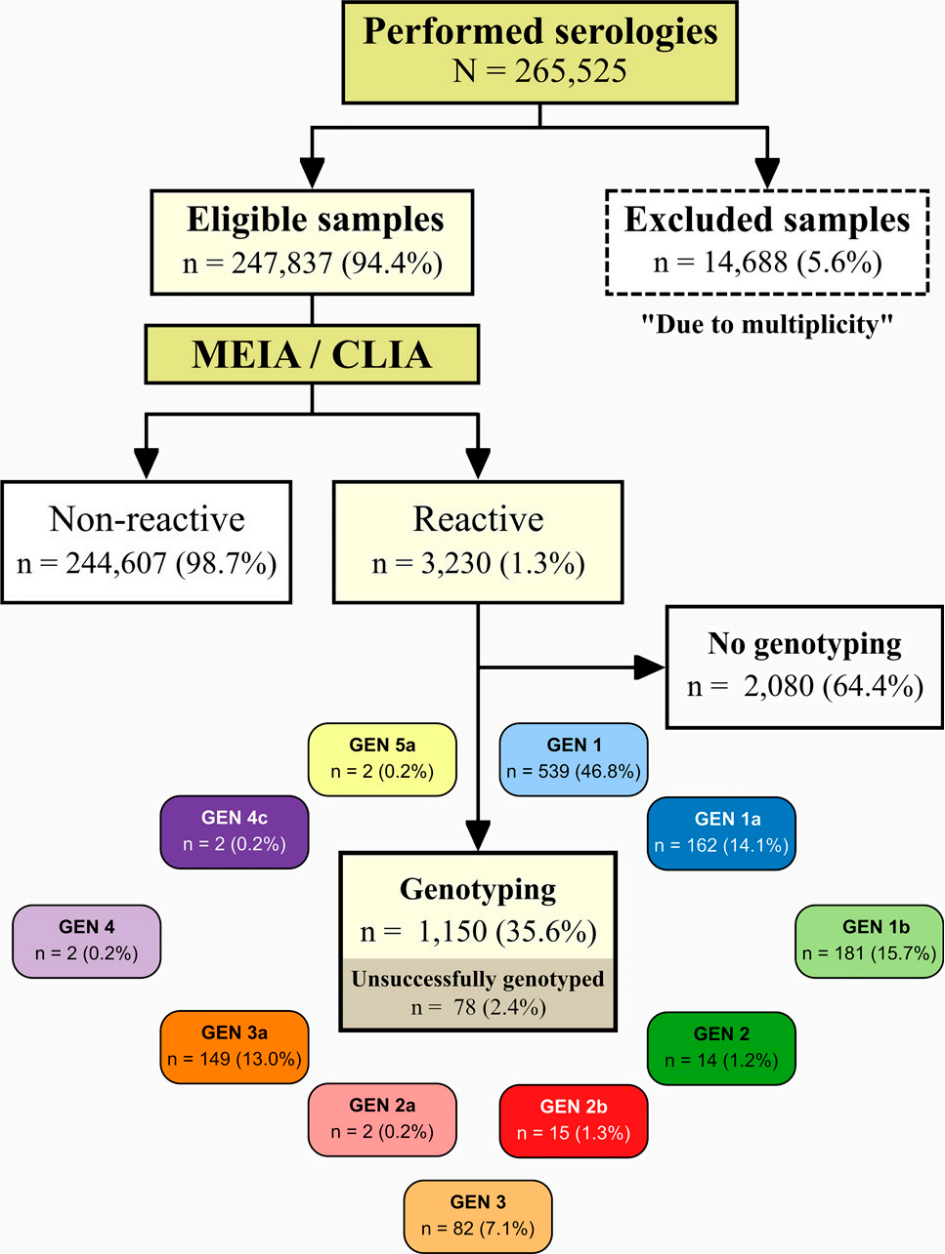
Flowchart of the study design. A genotyping analysis was performed for some of the samples (only when requested by the physician at the onset of treatment or if resistance was suspected). This figure appears in color at www.ajtmh.org.

A total of 247,837 samples were submitted for HCV serological analysis, with 94.7% (395/417) coverage of the municipalities throughout the state of Bahia (Figure 2). The median age of the study population was 31 years (IQR, 25–44 years), and the female:male ratio was 3.9:1.

**Figure 3.**
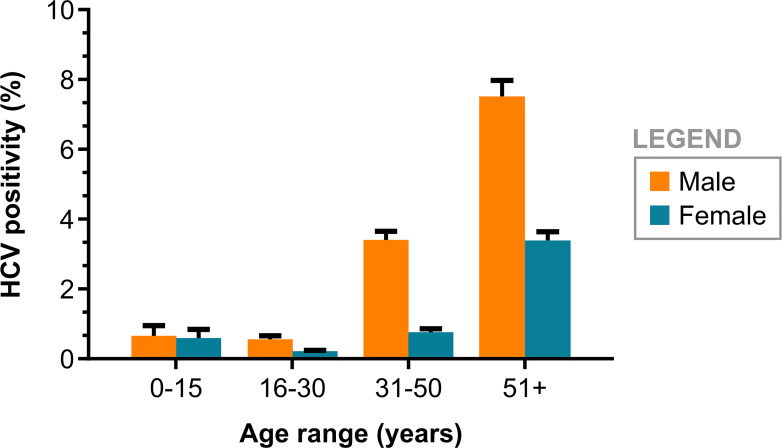
Anti-hepatitis C virus (anti-HCV) positivity among 45,199 males and 185,703 females according to age group in Bahia, Brazil, from 2004 to 2013. This figure appears in color at www.ajtmh.org.

The overall prevalence of anti-HCV in the sample was 1.3% (3,230/247,837; 95% CI: 1.26–1.35) (Figure 1). The HCV seroprevalence rates were 3.7% (1,865/50,430; 95% CI, 3.5–3.9) among males and 0.69% (1,365/197,407; 95% CI, 0.66–0.73) among females (*P* < 0.0001) (Table 1). The median age of the 197,407 females was 30.9 years (IQR, 24.6–39.9 years); however, it was 42.3 years (IQR, 30.0–58.0 years) for 50,430 males. For both sexes, the prevalence was found to increase significantly for individuals older than 31 years (males: *P* < 0.0001; females: *P* < 0.0001). Thirty individuals 15 years or younger presented positive serology for HCV, including three individuals younger than 4 years (Figure 3).

**Table 1:** Socio-demographic characteristics of individuals evaluated for hepatitis C virus in the state of Bahia, 2004–2013

Socio demographic characteristics		Study population	Individuals with anti-HCV reagent	*P* value
Sex, N (%)	Male	50,430 (20.3%)	1,865 (3.7%)	< 0.0001	
	Female	197,407 (79.7%)	1,365 (0.69%)		
Age, years	Mean (IQR)	41 (26–43)	55 (42–61)	< 0.0001	
Origin, N (%)	Salvador (capital)	53,974 (21.8%)	1,603 (49.6%)	< 0.0001	
	Others municipalities	193,863 (78.2%)	1,627 (50.4%)		

HCV = hepatitis C virus; IQR = interquartile range.

**Figure 4.**
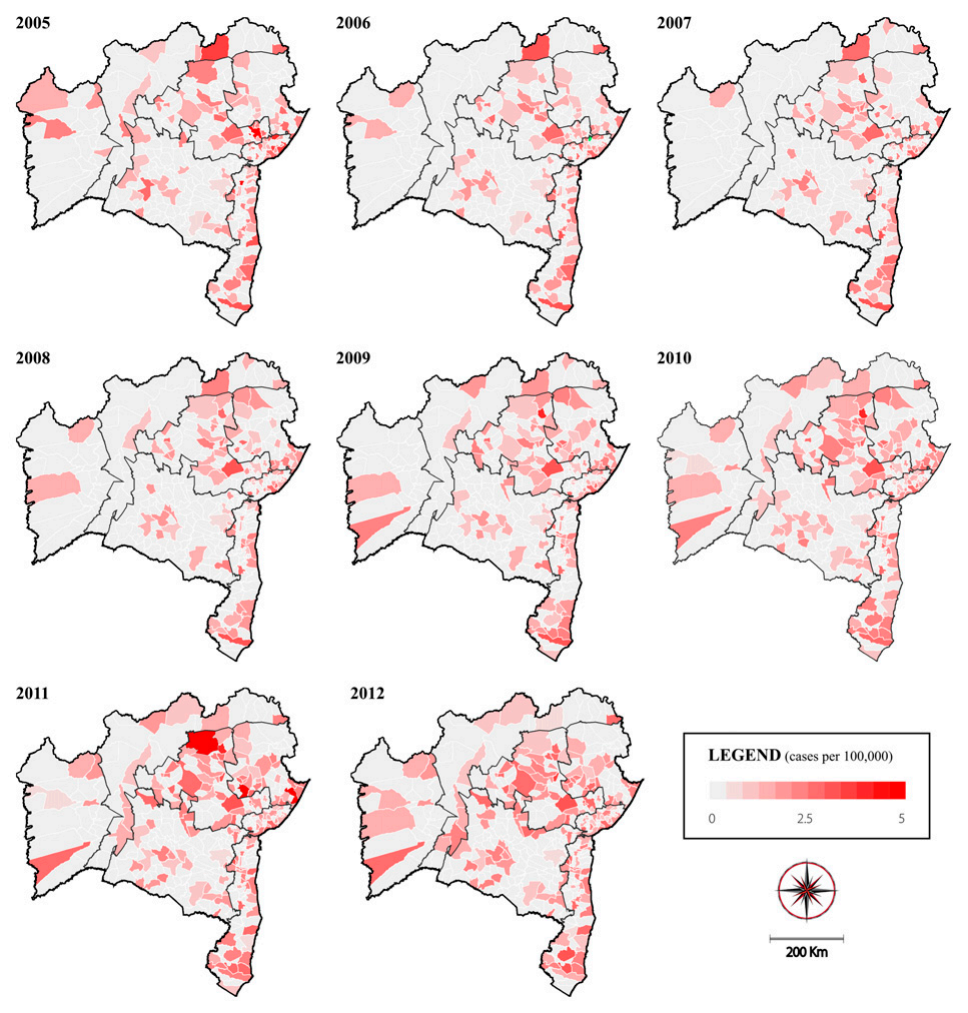
Spatiotemporal distribution of anti-hepatitis C virus (anti-HCV) positivity in the state of Bahia from 2004 to 2013, calculated using 3-year moving averages and considering the microregions or the state as units of analysis. This figure appears in color at www.ajtmh.org.

Of the municipalities that sent samples for evaluation, anti-HCV positivity was detected in 55.2% (218/395). The cumulative mean rate of positive HCV cases was 25.0 per 100,000 inhabitants in Bahia. Information regarding the city of origin was lacking for 0.7% of the anti-HCV-positive samples. Regarding the spatiotemporal distribution of HCV positivity in all microregions of Bahia at eight distinct time points (Figure 4), no difference was found in the number of positive cases, as evidenced by the first period of study (2005) during which 2.81 HCV cases per 100,000 inhabitants was found compared with 2.62 cases per 100,000 inhabitants during the final period (2012). However, an increase in the number of positive cases was observed, especially in the microregions of Senhor do Bonfim (0.17 cases per 100,000 inhabitants in 2005 versus 2.77 cases per 100,000 inhabitants in 2012) and Serrinha (0.45 cases per 100,000 inhabitants in 2005 versus 2.97 cases per 100,000 inhabitants in 2012).

In contrast, a decrease in the number of cases was observed in the microregions of Juazeiro (8.22 cases per 100,000 inhabitants in 2005 versus 0.29 cases per 100,000 inhabitants in 2012), Salvador (7.77 cases per 100,000 inhabitants in 2005 versus 4.79 cases per 100,000 inhabitants in 2012), Catu (2.78 cases per 100,000 inhabitants in 2005 versus 1.36 cases per 100,000 inhabitants in 2012), and Barreiras (2.45 cases per 100,000 inhabitants in 2005 versus 0.89 cases per 100,000 inhabitants in 2012).

**Figure 5.**
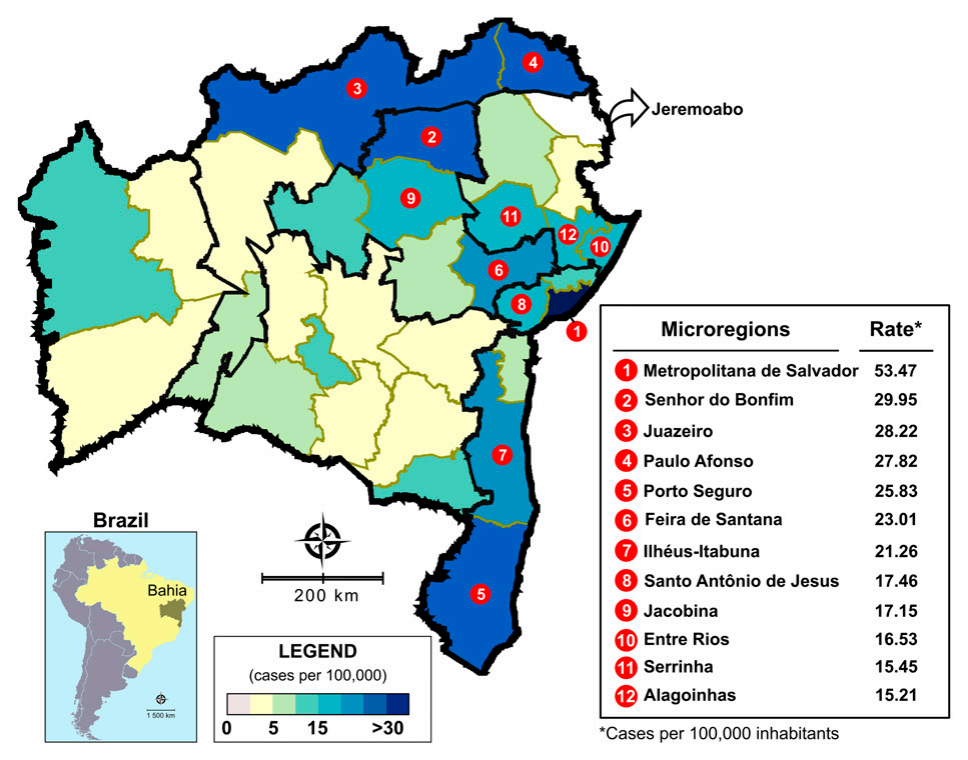
Spatial distribution of overall anti-hepatitis C virus (anti-HCV) positivity in the state of Bahia from 2004 to 2013, considering microregions as the unit of analysis. The 12 microregions with the highest rates of anti-HCV positivity per 100,000 inhabitants are highlighted. No information was retrieved from the Jeremoabo microregion (arrow). This figure appears in color at www.ajtmh.org.

Considering the grouping of anti-HCV-positive samples into microregions (Figure 5), seven areas demonstrated rates more than 20 HCV-positive cases per 100,000 inhabitants: Metropolitan Region of Salvador (53.47 cases per 100,000 inhabitants), Senhor do Bonfim (29.95 cases per 100,000 inhabitants), Juazeiro (28.22 cases per 100,000 inhabitants), Paulo Afonso (27.82 cases per 100,000 inhabitants), Porto Seguro (25.83 cases per 100,000 inhabitants), Feira de Santana (23.01 cases per 100,000 inhabitants), and Ilhéus-Itabuna (21.26 cases per 100,000 inhabitants). Rates of anti-HCV seroprevalence ranging from 15 to 20 cases per 100,000 inhabitants were also seen in five other microregions. In the remaining microregions, a homogenous distribution of anti-HCV positivity was observed. No information was retrieved from one microregion (Jeremoabo), however.

**Figure 6.**
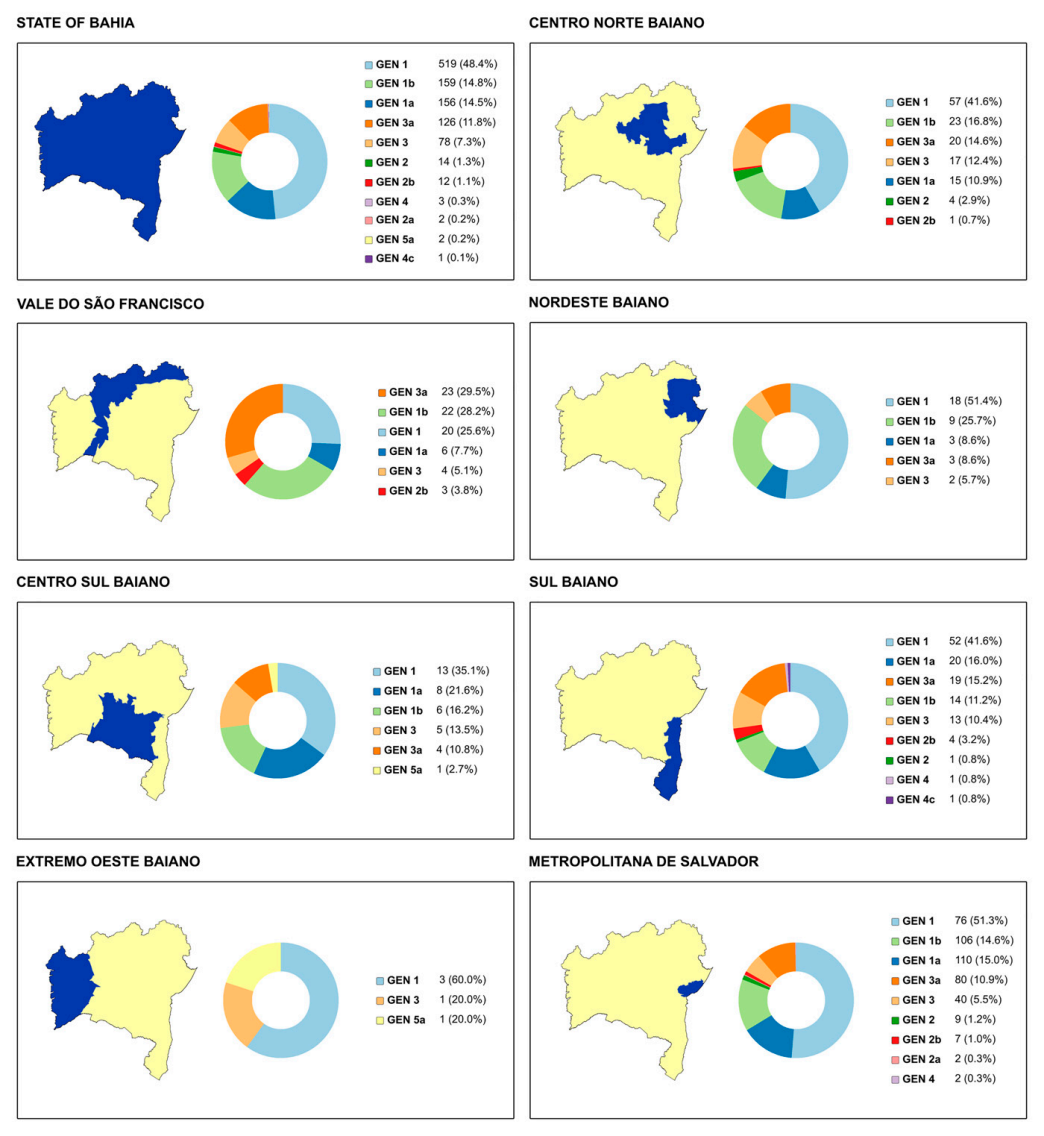
Distribution of hepatitis C virus (HCV) genotypes from 1,150 individuals infected in the mesoregions of the state of Bahia, Brazil, from 2004 to 2013. This figure appears in color at www.ajtmh.org.

The HCV genotype was determined for 1,150 (35.6%) of the 3,230 cases with anti-HCV antibodies. Seventy-eight samples were not successfully genotyped. Genotypes 1 (46.98%), 1a (14.1%), and 1b (15.7%) were the most frequently obtained, followed by genotypes 3a (13.0%), 3 (7.1%), 2 (1.2%), 2a (0.2%), and 2b (1.3%). Genotypes 4 (0.2%), 4c (0.2%), and 5a (0.2%) were less frequently identified. Some isolates could not be subtyped. Genotypes 1, 1a, 1b, 2, 2a, 2b, 3, and 3a were present among the seven mesoregions of the state, but genotype 4 was detected in mesoregions Metropolitan Region of Salvador and Sul Baiano. Genotype 5 was detected in the mesoregions of Centro Sul and Extremo Oeste (Figure 6).

## DISCUSSION

The data obtained during the present study demonstrate that the overall seroprevalence of HCV antibodies was 1.3% (3,230/247,837). The HCV infection rate found during this study is consistent with the prevalence (1.5%) of a population-based study in Salvador (the state capital)^19^ and that found during the national survey of viral hepatitis (1.38%) performed in the capitals of Brazil.^6^ However, this prevalence was higher than estimated for anti-HCV (0.94%) in the capitals of the northeast region.^6^ Prevalence studies evaluating the general population are rare. In Brazil, the prevalence of HCV ranges from 2.4% to 47% for vulnerable groups such as drug users (28.3%), individuals with renal failure (12.6%), individuals with coagulation disorders (47%), waste handlers (3.3%), and incarcerated individuals (2.4%).^8^^–^^10^^,^^12^^,^^20^ However, the prevalence of HCV is lower for sex workers and transgender women, and it remained less than 0.1% for blood donors and manual sugarcane cutters.^14^^,^^17^^,^^21^^,^^22^

There is no information regarding the geographical distribution of the anti-HCV seroprevalence in Bahia, which is the state with the largest population in the northeast and the fourth largest in Brazil. The present study demonstrates that individuals seropositive for anti-HCV antibodies were distributed throughout 31 of the 32 microregions of the state, thus corresponding to an overall rate of 21.2 cases per 100,000 individuals. Of note, the number of anti-HCV-positive cases in the state of Bahia remained similar throughout the study period (< 3 cases per 100,000 inhabitants). However, variations between microregions were observed. The microregions with the highest rates of anti-HCV antibodies were Ilhéus-Itabuna, Feira de Santana, Porto Seguro, Salvador, Jacobina, and Senhor do Bonfim, which are characterized as the great economic poles of the state because of their commercialism and tourism.

Notably, the city of Ipiaú, located in the Ilhéus-Itabuna microregion, had the highest anti-HCV rate per 100,000 inhabitants (112.04). We speculate that this high rate is associated with intravenous drug use as well as greater access to the diagnosis of HCV infection. Currently, the state of Bahia has 32 testing and counseling centers that provide screening for sexually transmitted infections and HCV. One of these testing and counseling centers is located in Ipiaú municipality. However, data regarding blood transfusion history and intravenous drug use were not available; therefore, it was not possible to estimate the probable route of infection. The incidence of transfusion-associated HCV was relatively high before the 1990s. Currently, injection drug use is considered the main route of HCV transmission in Brazil and the United States.^6^^,^^23^

Regarding the profile of HCV circulating genotypes in Bahia, the present study detected the presence of genotypes 1, 2, 3, 4, and 5. Genotype 1 and its subtypes (76.7%) were the most prevalent, followed by genotypes 3a (13.0%) and 3 (7.1%). It has been reported that HCV genotypes 1 and 3 are the most prevalent worldwide, accounting for approximately 46.2% and 30.1% of infections, respectively.^24^ Genotype 4 has been found most often in north Africa and the Middle East, whereas genotype 5 is more prevalent in South Africa.^25^^,^^26^ In relation to Brazil, genotype 1 is widely distributed in the different geographic regions of the country, genotype 2 is more frequent in the midwestern region, and 3 is more frequent in the southern region.^27^ Interestingly, in the present study, four cases of genotype 4 HCV infection were identified in the cities of Ipiaú, Teixeira de Freitas, and Salvador, and two genotype 5 cases were found in Vitória da Conquista and São Felix do Coribe. The presence of genotype 4 was first recorded in Brazil in the city of São Paulo in a patient who underwent kidney transplantation^28^; thereafter, it was found in an intravenous drug user in Salvador.^29^ HCV genotype 5 infections have been reported for only three individuals in the city of São Paulo in 2001.^30^

Regarding the demographic profile of individuals seropositive for anti-HCV antibodies in the present study, the average age was 55 years and most were males. However, the population evaluated during this study mainly comprised females. Interestingly, when the overall prevalence of anti-HCV antibodies was extrapolated to consider the entire population of the state of Bahia (i.e., all males and females), a similar prevalence (0.021%; 3,230 cases/15.2 million inhabitants) was found (0.03% versus 0.02%, respectively). Similar demographic profiles have been described in reports of studies conducted in other regions of Brazil and Bahia.^6^^,^^19^^,^^31^^,^^32^ In fact, it was expected that HCV infection would be more frequent in individuals 50 years or older because the laboratory diagnosis of HCV was adopted in 1989, and testing of blood donors became mandatory in Brazil only in 1993.^33^ Before this period, the sharing of syringes and the use of glass syringes were common practices and factors that contributed to HCV dissemination.^1^

In the present study, 3,230 cases of seropositive anti-HCV antibodies were diagnosed in 218 municipalities, thus corresponding to 52.2% of the municipalities of the state. From 2004 to 2013, the same period of this study, the state of Bahia registered 2,739 cases of HCV from 140 municipalities of the state in the Notifiable Disease Information System (SINAN).^18^ This could indicate the underreporting of HCV infection despite the notification of HCV infection being compulsory in Brazil since 1996. Considering the population of 15.2 million individuals in Bahia in 2015, 197,600 cases of HCV infection would be expected; of these, 138,320 would progress to chronic hepatitis (70% of cases), 27,664 (20%) would progress to cirrhosis, and 1,106 (4%) would progress to hepatocellular carcinoma.

This study was limited by nonrandomized sampling procedures and the predominant representation of females. Because the Brazilian Ministry of Health recommends screening pregnant women for sexually transmitted diseases, such as HIV and human T-lymphotropic virus, as well as hepatitis B virus and HCV, the Central Laboratory of Public Health of Bahia receives many samples from pregnant women, thus undoubtedly lending bias to the present results. Another limitation was that the presence of antibodies against HCV does not necessarily reflect active infection. Furthermore, of all samples with anti-HCV positivity, approximately 35% were submitted for PCR testing and approximately 33% were successfully genotyped. Our study was additionally limited by the lack of information regarding risk factors for HCV infection. However, regarding the representativeness of the municipalities, 94.7% of the state municipalities were evaluated throughout the study, thereby allowing us to evaluate the spatial distribution of HCV infection.

In conclusion, the results obtained during this study demonstrate that HCV was previously widespread throughout the state of Bahia. The predominance of males among anti-HCV seropositive subjects may reflect intravenous drug use in urban and rural areas. Studies evaluating the risk factors associated with the presence of HCV in these areas should be conducted to support more effective public prevention policies and identify patients who need treatment.
